# COVID-19 vaccine hesitancy: a midwifery survey into attitudes towards the COVID-19 vaccine

**DOI:** 10.1186/s12889-022-13540-y

**Published:** 2022-06-18

**Authors:** Funlayo Odejinmi, Rebecca Mallick, Christina Neophytou, Kade Mondeh, Megan Hall, Claire Scrivener, Katie Tibble, Mary Turay-Olusile, Nandita Deo, Doreen Oforiwaa, Rita Osayimwen

**Affiliations:** 1 Whipps Cross University Hospital, Barts Health NHS Trust, London, E11 1NR UK; 2 Princess Royal Hospital, Brighton and Sussex University Hospitals NHS Trust, Lewes Road, Haywards Heath, RH16 4EX UK; 3 Newham University Hospital, Barts Health NHS Trust, Glen Rd, London, E13 8S UK

**Keywords:** COVID-19, Vaccine, Racial disparity, Midwives, Ethnicity, Health equity

## Abstract

**Background:**

Ethnically minoritised people have been disproportionately affected by the COVID-19 pandemic. Emerging evidence suggests a lower uptake of the vaccine in ethnically minoritised people, particularly Black females of reproductive age. Unvaccinated pregnant women are high risk for morbidity and mortality from COVID-19. Midwives are the principal healthcare professionals responsible for counselling the pregnant population on decisions relating to vaccine uptake. The aim of this study was to explore midwifery uptake of and attitudes towards the COVID-19 vaccine in two ethnically diverse areas.

**Methods:**

A 45-point questionnaire was circulated over a six-week period to midwives employed in two teaching hospitals in England; London (Barts Health NHS Trust) and Sussex (Brighton and Sussex University Hospitals NHS Trust (BSUH)). A total of 378 out of 868 midwives responded. Results were analysed to determine vaccine uptake as well as factors influencing vaccine hesitancy and decision-making between the two trusts and ethnic groups. Thematic analysis was also undertaken.

**Results:**

Midwives of Black ethnicities were over 4-times less likely to have received a COVID-19 vaccine compared to midwives of White ethnicities (52% vs 85%, adjusted OR = 0.22, p = < 0.001). Overall, there were no significant differences between trusts in receipt of the COVID-19 vaccine (*p* = 0.13). Midwives at Barts Health were significantly more likely to have tested positive for COVID-19 compared to midwives at BSUH (adjusted OR = 2.55, *p* = 0.007). There was no statistical difference between ethnicities in testing positive for COVID-19 (*p* = 0.86). The most common concerns amongst all participants were regarding the long-term effect of the vaccine (35%), that it was developed too fast (24%), having an allergic reaction (22%) and concerns about fertility (15%). Amongst unvaccinated midwives, those of Black ethnicity had a higher occurrence of concern that the vaccine contained meat / porcine products (adjusted OR = 5.93, *p* = 0.04) and that the vaccine would have an adverse effect on ethnic minorities (adjusted OR = 4.42, *p* = 0.03).

**Conclusion:**

This study highlights the significantly higher level of vaccine hesitancy amongst Black midwives and offer insights into midwives’ concerns. This can facilitate future targeted public health interventions. It is essential that vaccine hesitancy amongst midwifery staff is addressed to improve vaccine uptake in the pregnant population.

## Background

Coronavirus disease 2019 (COVID-19) was first reported in December 2019 and was declared the latest global pandemic by the World Health Organisation (WHO) on the 19th of March 2020, which has led to severe morbidity and mortality worldwide [1, 2].

As reported by Public Health England (PHE) and the recent government white paper, ethnically minoritised people have been disproportionately affected by the pandemic [3]. This has been associated with occupational risk; ethnically minoritised people are more likely to be frontline workers, causing increased risk of exposure to COVID-19 and transmission to cohabiting family members [3–5]. In the first wave, Black and Asian healthcare staff accounted for 63% of deaths amongst health and social care workers, despite only making up 21% of the NHS workforce [6]. COVID-19 has also had a higher adverse effect for Black and ethnically minoritised pregnant women, with a UK study showing that 56% of 427 admissions due to COVID-19 in pregnancy were from Black or other ethnically minoritised groups [7].

There has been a rapid development of vaccines against SARS-CoV-2, some of which have utilised novel mRNA technology. Rollout began in the UK in December 2020, initially targeting the most vulnerable groups - including healthcare workers. Vaccine hesitancy, defined by the WHO as a delay in acceptance or refusal of safe vaccines despite availability, has been a major barrier in preventing severe infection with COVID-19 in the UK, particularly in the obstetric population [8]. Between July and September 2021, 1 in 6 patients who received extra corporeal membrane oxygenation were unvaccinated pregnant people [9].

General population data has shown vaccine uptake has been lowest in Black females of reproductive age [10–12], which is likely to compound the already 4 times higher risk of maternal mortality for in Black women in the UK [13]. Vaccine hesitancy may relate to several factors, including misinformation surrounding COVID-19 and distrust in the vaccination, healthcare providers and policy makers [14, 15]. Data has also shown higher rates of declining the vaccine amongst some health and social care workers. Those who felt pressure from their employer, and Black African and Mixed Black African healthcare workers were more likely to have declined the vaccine [15].

In November 2021 the Department of Health and Social Care amended legislation to make vaccination against COVID-19 mandatory for all patient-facing staff [16]. The Royal College of Nursing has stated that this risks further marginalising those who remain unvaccinated, rather than supporting them in accessing the vaccine [17].

Local data from Barts Health suggested that midwives were the least likely amongst healthcare staff to accept the COVID-19 vaccine. To explore this, a study was undertaken to determine vaccine uptake rates and explore the reasons for vaccine hesitancy amongst midwives in two UK National Health Service (NHS) trusts with differing demographics in the ethnicity of their workforce. The aim was to explore the factors that drive decision making regarding COVID-19 vaccination and to undertake a thematic analysis of midwifery views towards vaccine hesitancy.

## Methods

A mixed methods qualitative and quantitative survey of midwives employed in two large teaching hospitals, situated in London (Barts Health NHS Trust) and Sussex (Brighton and Sussex University Hospitals NHS Trust - BSUH) was undertaken. The questionnaire was initially developed using a small focus group consisting of midwives of differing experience at the Barts Health NHS site. The questionnaire consisting of 45 questions was circulated electronically to all midwives working in the two NHS Trusts over a six-week period. Midwives were asked whether they had received their COVID-19 vaccination, followed by a series of questions exploring the factors that influenced their decision and concerns regarding the COVID-19 vaccine. Demographics collected included ‘band’ which is a numerical system of nine progressive pay brackets covering all NHS staff except doctors, dentists, and senior managers, starting at Band 5 for newly qualified midwives. The survey was advertised via email, local trust bulletins and social media. Informed consent was obtained on filling the questionnaire. The data was collected on Microsoft Excel and analysed on both Microsoft Excel and SPSS (version 27). A minimum response rate of 30% was required for the validity of the observed differences.

Demographics were compared. Categorical variables with no natural ordering to the categories were compared between groups using the Chi-square test, or Fisher’s exact test if the numbers in some categories were small. Categorical variables with a natural ordering to the categories were compared between Trusts and Black/White ethnic groups using the Mann-Whitney test. The Kruskal-Wallis test was used to compare these factors between the three ethnic groups.

Subsequently the difference in outcomes between trusts and ethnicities was examined. Ethnicities included in the survey were grouped into broader categories for the analysis to allow adequate numbers for comparison. ‘Black or Black British – Caribbean’ and ‘Black or Black British – African’ were categorised as Black Ethnicities. ‘White British’, ‘White Irish’ and ‘any other White background’ were categorised as White Ethnicities. These two groups accounted for 333 out of 374 participants who responded to the question on ethnicity (89%). Remaining ethnicities were then similarly grouped into Asian, Mixed Race or Other. Due to relatively small numbers of participants from each of these groups, only comparison between grouped White Ethnicities and Black Ethnicities was presented in the analysis. For each outcome analyses were performed with and without adjustment for potentially confounding variables. Analysis of COVID-19 vaccine acceptance and related outcomes and were all binary in nature (either yes or no). Therefore, logistic regression analysis and multiple logistic regression analysis were carried out.

The two trusts varied statistically in terms of ethnicity (p = < 0.001) and age (*p* = 0.01). Some difference in years experience was also noted between trust (*p* = 0.18). Therefore, comparisons between trusts were adjusted for ethnic group, age, and level of experience. There was no significant difference between trust in gender (*p* = 1.00), band (*p* = 0.57), and general health (*p* = 0.54) and so these variables were not adjusted for.

With regards to examining outcomes between Black and White ethnic groups, there was a significant difference in age (p = < 0.001), experience (*p* = 0.008) and band (*p* = 0.08). We therefore adjusted comparisons between ethnicity for these variables. We did not adjust for gender (*p* = 0.57) and general health (*p* = 0.53) as these variables were not statistically significant.

Outcomes are expressed as odds ratios and presented with corresponding confidence intervals. To minimise confounding, only adjusted odds ratios (aOR) are discussed in the results.

Other outcomes related to concerns about the vaccine. Midwives were given three outcome options as to their level of concern: no, don’t know, yes. These were all assumed to be ordinal outcome, and thus the analyses were performed using ordinal logistic regression. Concerns were presented as raw data from all participants to ascertain which concerns were most prevalent amongst all midwives. Outcomes were then analysed in participants who were unvaccinated, to focus the findings on concerns which drove vaccine hesitancy. The same approach to the analyses was made as to that described for the binary outcomes, and comparisons between trust and ethnicity were adjusted for the same variables.

Lastly, we performed a thematic analysis of the qualitative data relating to responses to two open questions regarding midwives’ personal views on the COVID-19 vaccine, and what they believe would increase vaccine uptake.

## Results

A total of 378 midwives responded from both teaching hospitals: 228 out of 580 at Barts Health and 150 out of 288 at BSUH, translating to a respondent rate of 44% overall; 39 and 52% respectively. Across both trusts 282 midwives had accepted the COVID-19 vaccine (75%).

Table 1 summarises the demographic characteristics of the midwives in the two trusts. Over a third of Barts staff were of Black ethnic groups, compared to no staff from BSUH. Midwives from Barts Health were generally younger, with 50% aged under 40, compared to only 36% from BSUH.

**Table 1:** Demographics by Trust

Factor	Category	BSUH [***N*** = 150]		Barts [***N*** = 228]		***P***-value
		n	n (%)	n	n (%)	
Age	< 25	150	4 (3%)	228	20 (9%)	**0.01**
	25–29		10 (7%)		38 (17%)	
	30–34		19 (13%)		29 (13%)	
	35–39		19 (13%)		26 (11%)	
	40–44		28 (19%)		15 (7%)	
	45–49		17 (11%)		29 (13%)	
	50–54		23 (15%)		31 (14%)	
	55–59		25 (17%)		29 (13%)	
	≥ 60		5 (3%)		11 (5%)	
Gender	Female	149	148 (99%)	226	224 (99%)	1.00
	Male		1 (1%)		2 (1%)	
Ethnic Group	White	150	145 (97%)	224	108 (48%)	**< 0.001**
	Black		0 (0%)		80 (36%)	
	Asian		1 (1%)		12 (5%)	
	Mixed race		4 (3%)		15 (7%)	
	Other		0 (0%)		9 (4%)	
Band	Band 5	150	14 (9%)	223	27 (12%)	0.57
	Band 6		96 (64%)		127 (57%)	
	Band 7		36 (24%)		52 (23%)	
	Band 8		4 (3%)		17 (8%)	
Experience	0–2 years	150	18 (12%)	227	46 (21%)	**0.18**
	3–5 years		28 (19%)		33 (15%)	
	6–10 years		32 (21%)		43 (19%)	
	10–15 years		21 (14%)		43 (19%)	
	> 15 years		51 (34%)		62 (27%)	
General health	Poor / fair	149	9 (6%)	226	12 (5%)	0.54
	Good		35 (23%)		61 (27%)	
	Very good		70 (47%)		83 (37%)	
	Excellent		35 (23%)		70 (31%)	

A summary of comparisons of the demographic between Black and White ethnic groups is shown in Table 2. White midwives were typically younger than Black midwives: almost half (46%) of White midwives were aged under 40, compared to 24% of Black midwives. Probably associated with age, Black midwives were more likely to have over 10 years experience and be higher band. Over two thirds (68%) of Black midwives had at least 10 years of experience compared to only 43% of White midwives.

**Table 2:** Demographics by Ethnicity

Factor	Category	White [***N*** = 253]		Black [***N*** = 80]		Other [***N*** = 41]		***P***-value	
		n	n (%)	n	n (%)	n	n (%)	Overall ^(*)^	B vs. W ^(**)^
Age	< 25	253	11 (4%)	80	3 (4%)	41	9 (22%)	**< 0.001**	**< 0.001**
	25–29		36 (14%)		4 (5%)		8 (20%)		
	30–34		36 (14%)		4 (5%)		7 (17%)		
	35–39		35 (14%)		8 (10%)		2 (5%)		
	40–44		29 (11%)		12 (15%)		2 (5%)		
	45–49		30 (12%)		10 (13%)		5 (12%)		
	50–54		33 (13%)		19 (24%)		2 (5%)		
	55–59		33 (13%)		17 (21%)		4 (10%)		
	≥ 60		10 (4%)		3 (4%)		2 (5%)		
Gender	Female	251	249 (99%)	80	79 (99%)	41	41 (100%)	0.69	0.57
	Male / Non-Binary		2 (1%)		1 (1%)		0 (0%)		
Band	Band 5	251	27 (11%)	79	7 (9%)	40	7 (18%)	0.08	0.08
	Band 6		154 (61%)		43 (54%)		24 (60%)		
	Band 7		60 (24%)		19 (24%)		8 (20%)		
	Band 8		10 (4%)		10 (13%)		1 (3%)		
Experience	0–2 years	253	40 (16%)	80	9 (11%)	40	14 (35%)	**< 0.001**	**0.008**
	3–5 years		47 (19%)		8 (10%)		5 (13%)		
	6–10 years		56 (22%)		9 (11%)		10 (25%)		
	10–15 years		36 (14%)		24 (30%)		3 (8%)		
	> 15 years		74 (29%)		30 (38%)		8 (20%)		
General health	Poor / fair	252	15 (6%)	79	5 (6%)	41	1 (5%)	0.69	0.53
	Good		61 (24%)		19 (24%)		15 (37%)		
	Very good		110 (44%)		29 (37%)		14 (34%)		
	Excellent		66 (26%)		26 (33%)		11 (27%)		

(*) *P*-values indicating the significance of the overall difference between the three ethnic groups

(**) *P*-values indicating the significance of the difference between Black and White midwives

Table 3 summarises the results on previous COVID-19 infection and vaccination uptake rates between midwives from the two trusts.

**Table 3:** COVID-19 and vaccination outcomes by Trust

Factor	Category	BSUH [*N* = 150]		Barts [*N* = 228]		Unadjusted		Adjusted ^b^	
		n	n (%)	n	n (%)	OR (95% CI) ^a^	*P*-value	OR (95% CI) ^a^	*P*-value
Opposition to flu vaccine	No	148	121 (82%)	226	166 (73%)	1.61 (0.97, 2.70)	0.06	1.16 (0.59, 2.28)	0.67
	Yes		27 (18%)		60 (27%)				
Received COVID vaccine	No	149	19 (13%)	226	74 (33%)	0.30 (0.17, 0.52)	**< 0.001**	0.55 (0.27, 1.12)	0.10
	Yes		130 (87%)		152 (67%)				
Able to self-isolate	No	149	70 (47%)	224	105 (47%)	1.00 (0.66, 1.52)	0.98	1.33 (0.76, 2.31)	0.32
	Yes		79 (53%)		119 (53%)				
Tested COVID positive in past	No	149	129 (87%)	226	161 (71%)	2.60 (1.50, 4.52)	**0.001**	2.55 (1.30, 5.01)	**0.007**
	Yes		20 (13%)		65 (29%)				

^a^ Odds ratios expressed as odds of a yes outcome for Barts staff relative to odds for BSUH staff

^b^ Trust differences adjusted for: age, experience and ethnicity

There was no significant difference between trusts in receipt of the COVID-19 vaccine (aOR = 0.55, *p* = 0.10). Midwives at Barts Health were significantly more likely to have tested positive for COVID-19 compared to midwives at BSUH (aOR = 2.55, *p* = 0.007).

The same outcomes were compared between ethnicities as shown in Table 4. Although there was no statistical difference between ethnicities in testing positive for COVID-19 (aOR = 1.07, *p* = 0.86), midwives of Black ethnicities were over 4 times less likely to have had the COVID-19 vaccine compared to midwives of White ethnicities (52% vs 85%, aOR = 0.22, *p* = <0.001). The two groups did not significantly vary in terms of their ability to self-isolate (aOR = 0.61, *p* = 0.16).

**Table 4:** COVID-19 and Vaccination outcomes by Ethnicity (White and Black ethnicities only)

Factor	Category	White [*N* = 253]		Black [*N* = 80]		Unadjusted		Adjusted ^b^	
		n	n (%)	n	n (%)	OR (95% CI) ^a^	*P*-value	OR (95% CI) ^a^	*P*-value
Opposition to flu vaccine	No	251	205 (82%)	79	46 (58%)	3.20 (1.85, 5.54)	**< 0.001**	2.87 (1.38, 5.97)	**0.005**
	Yes		46 (18%)		33 (42%)				
Received COVID vaccine	No	252	37 (15%)	79	38 (48%)	0.19 (0.11, 0.33)	**< 0.001**	0.22 (0.11, 0.46)	**< 0.001**
	Yes		215 (85%)		41 (52%)				
Able to self-isolate	No	251	113 (45%)	78	38 (49%)	0.86 (0.52, 1.43)	0.57	0.61 (0.31, 2.21)	0.16
	Yes		138 (55%)		40 (51%)				
Tested COVID positive in past	No	252	202 (80%)	79	57 (72%)	1.56 (0.87, 2.79)	0.13	1.07 (0.51, 2.24)	0.86
	Yes		50 (20%)		22 (28%)				

^a^ Odds ratios expressed as odds of a yes outcome for Black midwives relative to odds for White midwives

^b^ Ethic differences adjusted for: age, band, experience and Trust

As part of the survey participants were asked whether they were opposed to the flu vaccine. There was no significant difference between trusts for opposition to the flu vaccine (aOR = 1.16, *p* = 0.67). Black midwives had nearly 3 times the odds of opposing influenza vaccination compared to White midwives (aOR = 2.87, *p* = 0.005).

**Fig. 1:**
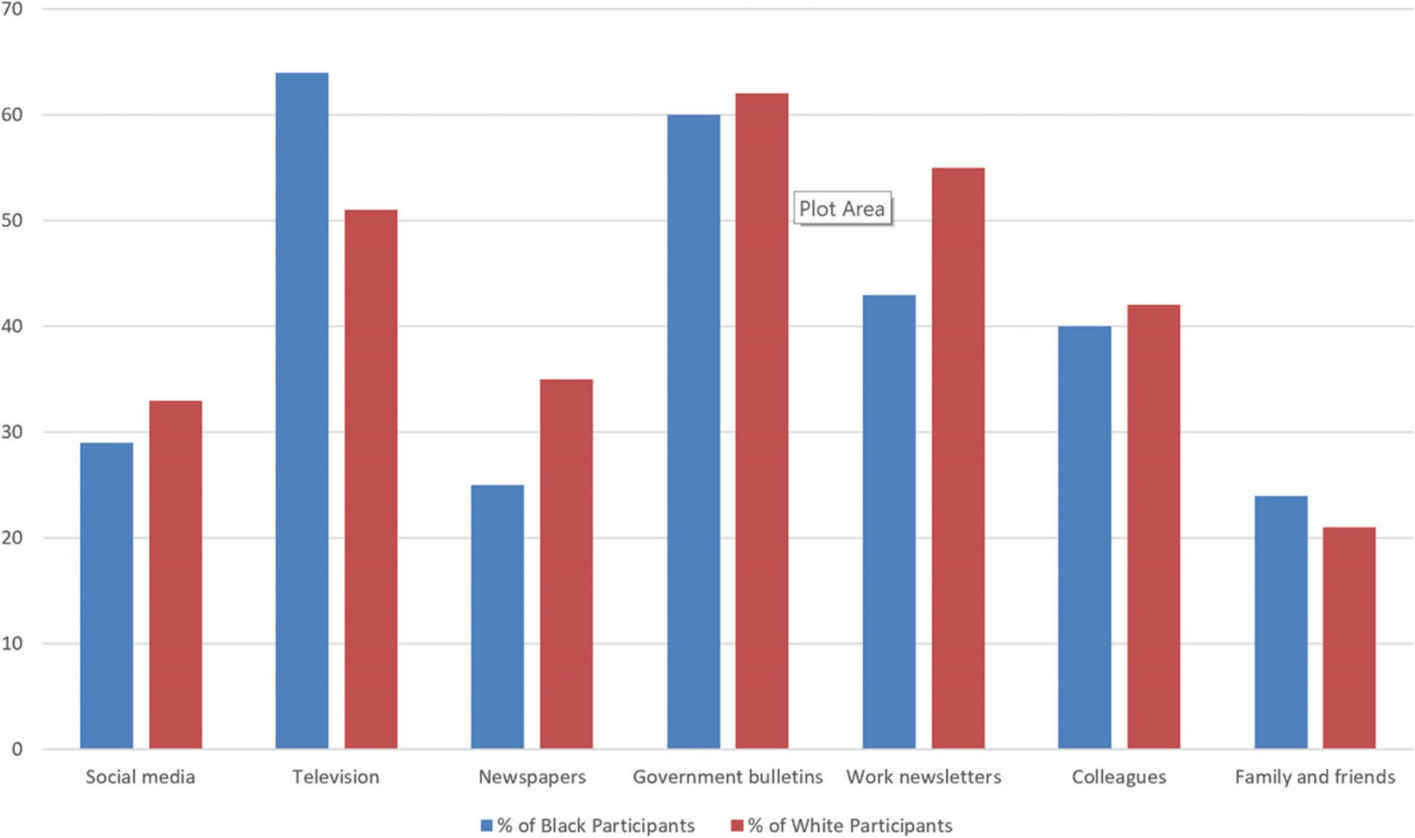
Graph comparing main source of information on the COVID-19 vaccine between Black and White ethnic groups

Figure 1 compares where midwives accessed information regarding the COVID-19 vaccine between ethnic groups. The most frequent information sources used by both ethnic groups were government bulletins (Black = 60%, White = 62%), television (Black = 63%, White = 50%), and work newsletters (Black = 43%, White = 55%).

**Fig. 2:**
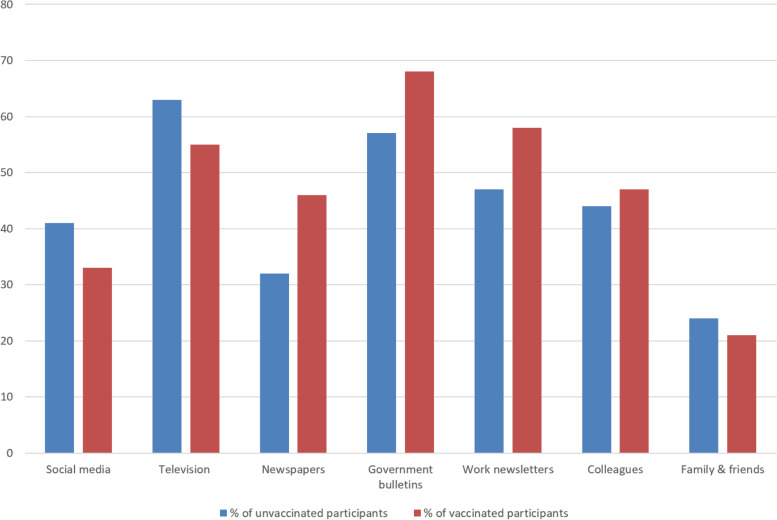
Graph comparing main source of information on the COVID-19 between unvaccinated and vaccinated participants

Figure 2 compares where midwives access information on the vaccine by vaccination status. There was no strong evidence that any of the information sources varied between the two groups. There was slight evidence that unvaccinated staff were less likely to get information from government bulletins (*p* = 0.07) or work newsletters (p = 0.07) and more likely to get information from family and friends (*p* = 0.09). However, none of these results quite reached statistical significance.

Table 5 presents the concerns asked about in the survey, and the percentage of midwives which answered yes to each concern. The most common concerns about the COVID-19 vaccine were concerns about long-term effects (35%), that the vaccine was developed too fast (24%), concerns about having an allergic reaction (24%) and concerns about fertility (15%).

**Table 5:** Vaccine concerns -answer ‘yes’ to concern in all participants

Concern	[*N* = 378] n (%)
Long term effects	133 (35%)
Allergic reaction	85 (22%)
Interfere with genetic code	22 (5%)
Developed too fast	93 (24%)
Government able to track you	13 (3%)
Interfere with Fertility	59 (15%)
Made of porcine/meat products	9 (2%)
Contains fetal tissue	25 (6%)
Adverse effect on ethnic minorities	28 (7%)
Get coronavirus from vaccine	18 (4%)

Tables 6 and 7 present a comparison of concerns relating to the COVID-19 vaccine in unvaccinated participants, between the two trusts. After adjustment in the regression models, all concerns were no longer significantly different between trusts. There was no significant difference in awareness of the Tuskegee Syphilis Trial between trusts (aOR = 1.09, *p* = 0.86). When asked if they would prefer a choice in the type of vaccine received similar numbers at both sites answered in the positive, 70 and 67% at Barts Health and BSUH respectively (*p* = 0.572).

**Table 6:** Vaccine concerns in unvaccinated staff by Trust (part 1)

Concern	Category	BSUH [*N* = 19]		Barts [*N* = 74]		Unadjusted		Adjusted ^b^	
		n	n (%)	n	n (%)	OR (95% CI) ^a^	*P*-value	OR (95% CI) ^a^	*P*-value
Long term effects	No	18	5 (28%)	61	8 (13%)	1.51 (0.46, 4.96)	0.50	1.17 (0.26, 5.24)	0.84
	Don’t know		0 (0%)		6 (10%)				
	Yes		13 (72%)		47 (77%)				
Allergic reaction	No	18	12 (67%)	61	18 (30%)	4.48 (1.49, 13.4)	**0.007**	2.72 (065, 11.4)	0.17
	Don’t know		1 (6%)		6 (10%)				
	Yes		5 (28%)		37 (61%)				
Interfere with genetic code	No	18	14 (78%)	61	32 (52%)	3.29 (0.99, 11.0)	0.05	3.88 (0.77, 19.8)	0.10
	Don’t know		3 (17%)		16 (26%)				
	Yes		1 (6%)		13 (21%)				
Developed too fast	No	18	7 (39%)	61	11 (18%)	4.37 (1.62, 11.8)	**0.003**	2.57 (0.69, 9.66)	0.16
	Don’t know		7 (39%)		10 (16%)				
	Yes		4 (22%)		40 (66%)				
Government able to track you	No	18	16 (89%)	61	38 (62%)	5.06 (1.07, 23.9)	**0.04**	4.62 (0.74, 29.0)	0.10
	Don’t know		2 (11%)		15 (25%)				
	Yes		0 (0%)		8 (13%)				
Interfere with fertility	No	18	10 (56%)	61	19 (31%)	2.77 (1.00, 7.63)	0.05	1.81 (0.47, 7.00)	0.39
	Don’t know		4 (22%)		15 (25%)				
	Yes		4 (22%)		27 (44%)				

^a^ Odds ratios expressed as odds of next highest outcome category for Barts staff relative to odds for BSUH staff

^b^ Trust differences adjusted for: age, experience and ethnicity

**Table 7:** Vaccine concerns in unvaccinated staff by Trust (part 2)

Concern	Category	BSUH [*N* = 19]		Barts [*N* = 74]		Unadjusted		Adjusted ^b^	
		n	n (%)	n	n (%)	OR (95% CI) ^a^	*P*-value	OR (95% CI) ^a^	*P*-value
Made of porcine /meat products	No	18	15 (83%)	61	41 (67%)	2.37 (0.62, 9.10)	0.21	0.58 (0.09, 3.71)	0.57
	Don’t know		2 (11%)		15 (25%)				
	Yes		1 (5%)		5 (8%)				
Contains fetal tissue	No	18	14 (78%)	61	34 (56%)	2.96 (0.89, 9.87)	0.08	3.18 (0.67, 15.1)	0.15
	Don’t know		3 (17%)		13 (21%)				
	Yes		1 (6%)		14 (23%)				
Adverse effect on ethnic minorities	No	18	10 (56%)	61	14 (23%)	4.58 (1.60, 13.1)	**0.005**	1.72 (0.43, 6.87)	0.47
	Don’t know		7 (39%)		29 (48%)				
	Yes		1 (6%)		18 (30%)				
Get coronavirus from vaccine	No	18	16 (89%)	61	42 (69%)	3.35 (0.70, 16.1)	0.13	3.00 (0.40, 22.7)	0.29
	Don’t know		0 (0%)		8 (13%)				
	Yes		2 (11%)		11 (18%)				

^a^ Odds ratios expressed as odds of next highest outcome category for Barts staff relative to odds for BSUH staff

^b^ Trust differences adjusted for: age, experience and ethnicity

Tables 8, 9 and 10 present a comparison of concerns relating to vaccine hesitancy between unvaccinated midwives of Black and White ethnicities. After adjustment, only two concerns differed significantly between ethnicities. Black midwives had almost 6 times the occurrence of concern that the vaccine contained porcine / meant products (aOR = 5.93, *p* = 0.04), and 4 times the concern that the vaccine would have an adverse effect on ethnic minorities (aOR = 4.42, *p* = 0.03).

**Table 8:** Vaccine concerns in unvaccinated staff by Ethnicity (White and Black ethnicities only) (part 1)

Concern	Category	White [*N* = 37]		Black [*N* = 38]		Unadjusted		Adjusted ^b^	
		n	n (%)	N	n (%)	OR (95% CI) ^a^	*P*-value	OR (95% CI) ^a^	*P*-value
Long term effects	No	34	9 (26%)	31	2 (6%)	2.77 (0.84, 9.11)	0.09	1.66 (0.36, 7.66)	0.51
	Don’t know		2 (6%)		3 (10%)				
	Yes		23 (68%)		26 (84%)				
Allergic reaction	No	34	19 (56%)	31	7 (23%)	3.83 (1.41, 10.4)	**0.009**	2.04 (0.53, 7.89)	0.30
	Don’t know		2 (6%)		3 (10%)				
	Yes		13 (38%)		21 (68%)				
Interfere with genetic code	No	34	25 (74%)	31	15 (48%)	2.12 (0.78, 5.81)	0.14	0.78 (0.19, 3.23)	0.74
	Don’t know		2 (6%)		12 (39%)				
	Yes		7 (21%)		4 (13%)				
Developed too fast	No	34	11 (32%)	31	5 (16%)	2.90 (1.09, 7.69)	**0.03**	2.12 (0.54, 8.35)	0.28
	Don’t know		9 (26%)		5 (16%)				
	Yes		14 (41%)		21 (68%)				
Government able to track you	No	34	26 (76%)	31	17 (55%)	2.76 (0.97, 7.86)	0.06	0.97 (0.24, 3.84)	0.97
	Don’t know		6 (18%)		9 (29%)				
	Yes		2 (6%)		5 (16%)				
Interfere with fertility	No	34	18 (53%)	31	9 (29%)	2.99 (1.17, 7.65)	**0.02**	2.57 (0.69, 9.52)	0.16
	Don’t know		8 (24%)		7 (23%)				
	Yes		8 (24%)		15 (48%)				

^a^ Odds ratios expressed as odds of next highest outcome category for Black midwives relative to odds for White midwives

^b^ Ethnic differences adjusted for: age, band, experience and Trust

**Table 9:** Vaccine concerns by Ethnicity (White and Black ethnicities only) (part 2)

Concern	Category	White [*N* = 253]		Black [*N* = 80]		Unadjusted		Adjusted ^b^	
		n	n (%)	n	n (%)	OR (95% CI) ^a^	*P*-value	OR (95% CI) ^a^	*P*-value
Made of porcine / meat products	No	242	224 (93%)	69	47 (68%)	5.65 (2.82, 11.3)	**< 0.001**	6.31 (2.46, 16.2)	**< 0.001**
	Don’t know		14 (6%)		19 (28%)				
	Yes		4 (2%)		3 (4%)				
Contains fetal tissue	No	242	207 (86%)	69	40 (58%)	4.20 (2.34, 7.54)	**< 0.001**	3.54 (1.63, 7.68)	**0.001**
	Don’t know		22 (9%)		18 (26%)				
	Yes		13 (5%)		11 (16%)				
Adverse effect on ethnic minorities	No	242	168 (69%)	69	29 (42%)	3.47 (2.04, 5.92)	**< 0.001**	3.85 (1.96, 7.52)	**< 0.001**
	Don’t know		65 (27%)		27 (39%)				
	Yes		9 (4%)		13 (19%)				
Get coronavirus from vaccine	No	242	230 (95%)	69	52 (75%)	5.96 (2.69, 13.2)	**< 0.001**	2.42 (0.90, 6.50)	0.08
	Don’t know		3 (1%)		10 (14%)				
	Yes		9 (4%)		7 (10%)				

^a^ Odds ratios expressed as odds of next highest outcome category for Black midwives relative to odds for White midwives

^b^ Ethnic differences adjusted for: age, band, experience and Trust

**Table 10:** Vaccine concerns in unvaccinated staff by Ethnicity (White and Black ethnicities only) (part 2)

Concern	Category	White [*N* = 37]		Black [*N* = 38]		Unadjusted		Adjusted ^b^	
		n	n (%)	n	n (%)	OR (95% CI) ^a^	*P*-value	OR (95% CI) ^a^	*P*-value
Made of porcine / meat products	No	34	30 (88%)	31	18 (58%)	5.14 (1.46, 18.1)	**0.01**	5.93 (1.06, 33.1)	**0.04**
	Don’t know		2 (6%)		10 (32%)				
	Yes		2 (6%)		3 (10%)				
Contains fetal tissue	No	34	23 (68%)	31	15 (48%)	2.32 (0.87, 6.16)	0.09	1.30 (0.35, 4.83)	0.69
	Don’t know		6 (18%)		7 (23%)				
	Yes		5 (15%)		9 (29%)				
Adverse effect on ethnic minorities	No	34	18 (53%)	31	3 (10%)	6.37 (2.29, 17.7)	**< 0.001**	4.42 (1.17, 16.7)	**0.03**
	Don’t know		12 (35%)		18 (58%)				
	Yes		4 (12%)		10 (32%)				
Get coronavirus from vaccine	No	34	28 (82%)	31	20 (65%)	2.14 (0.69, 6.66)	0.19	2.19 (2.23, 6.19)	0.84
	Don’t know		0 (0%)		6 (19%)				
	Yes		6 (18%)		5 (16%)				

^a^ Odds ratios expressed as odds of next highest outcome category for Black midwives relative to odds for White midwives

^b^ Ethnic differences adjusted for: age, band, experience and Trust

More Black midwives were aware of the Tuskegee Syphilis Trial compared to White midwives (aOR = 6.96, p = < 0.001). When asked if they would prefer a choice in the type of vaccine, there was no significant difference between the two ethnicities with both groups answering in the positive, 72 and 65% amongst Black and White midwives respectively (*p* = 0.572).

**Fig. 3:**
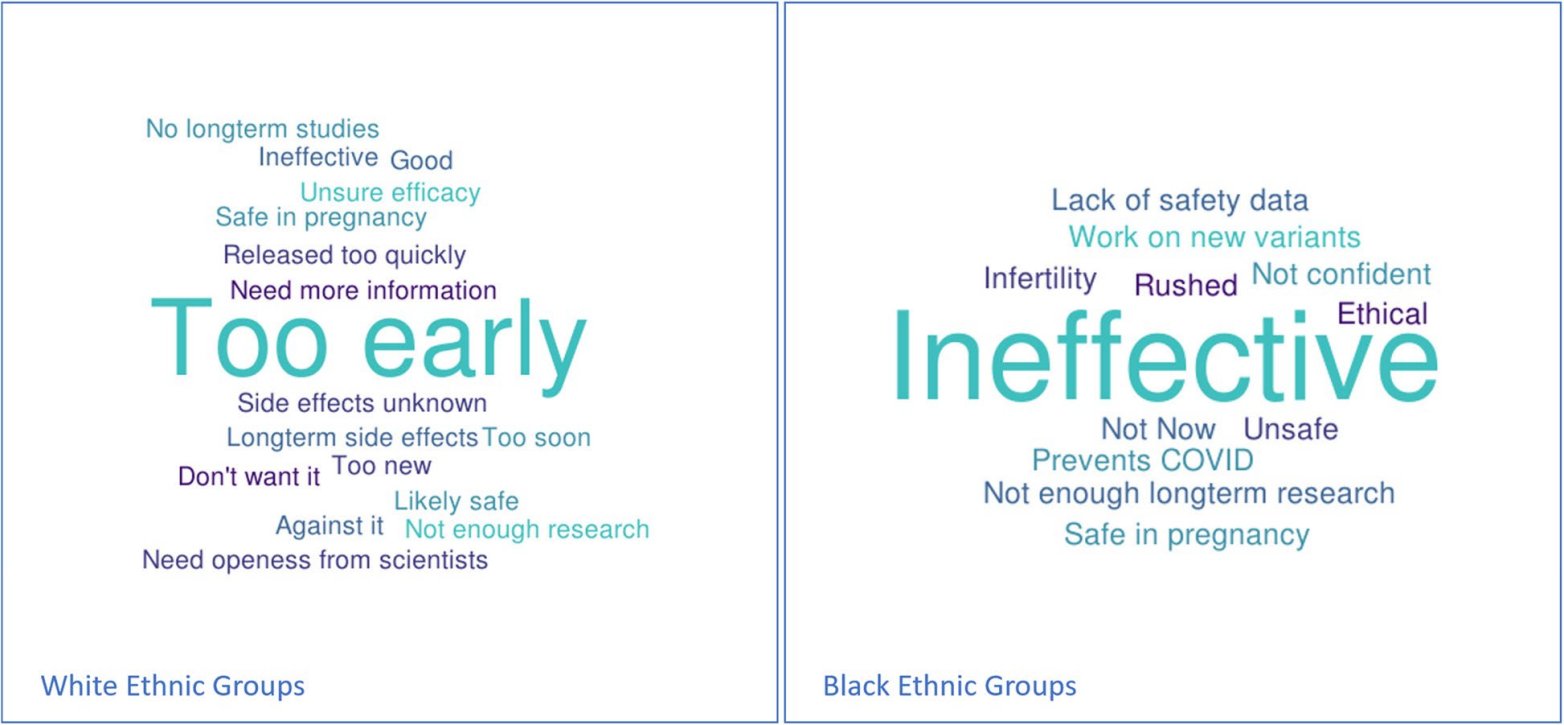
Word Cloud showing responses of unvaccinated participants to the question ‘What are your personal views on the COVID-19 Vaccine? Separated by Ethnicity

**Fig. 4:**
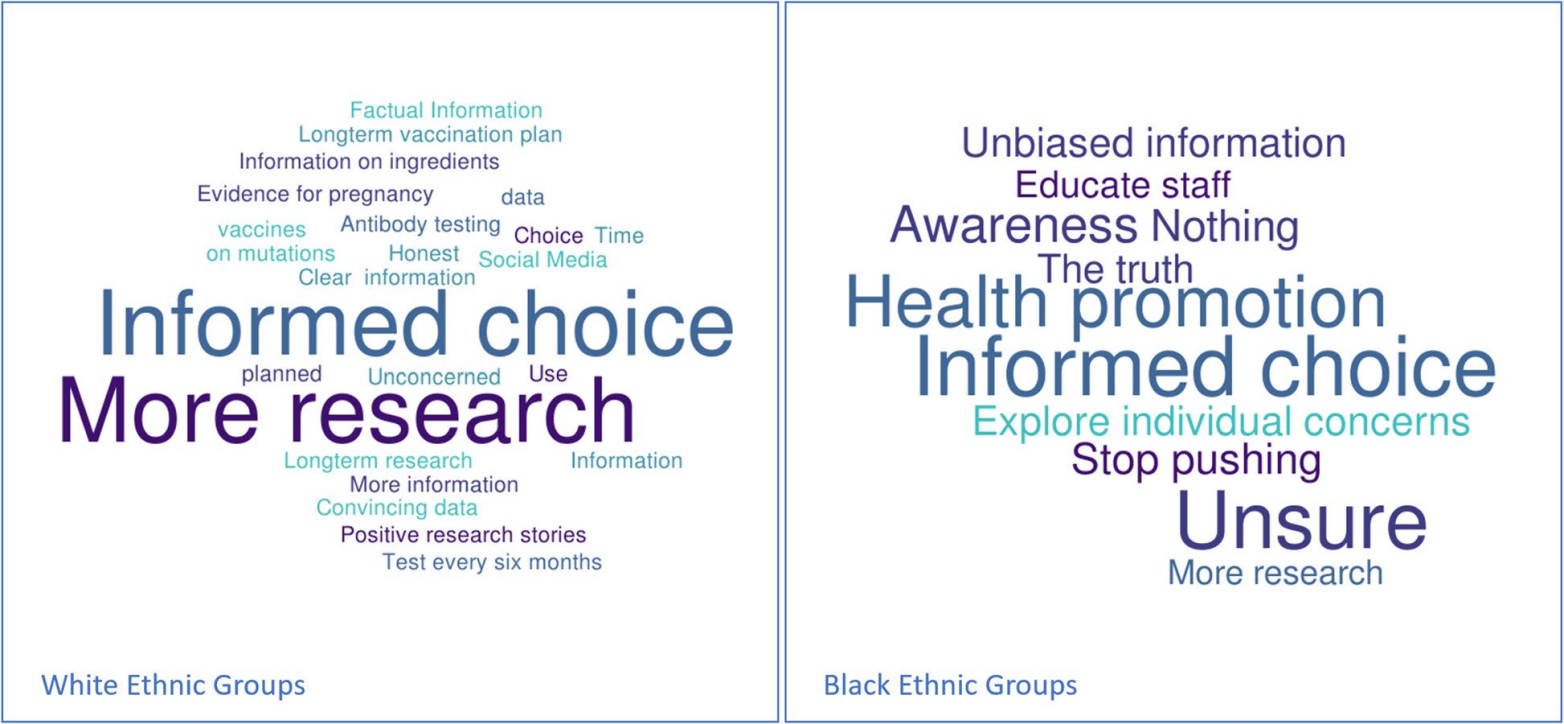
Word Cloud showing responses of unvaccinated participants to the question ‘What do you think would be useful to increase the uptake of COVID-19 vaccinations?’ Separated by ethnicity

Thematic analysis of the qualitative data collected from unvaccinated participants was undertaken and is summarised in Figs. 3 and 4.

With regards to respondents’ personal views on the COVID-19 vaccine, midwives were predominantly positive across both sites, expressing happiness that the vaccine had been introduced and hope that it would be the solution to easing of restrictions and provide protection for vulnerable groups and their families.

The most common themes amongst those who were not vaccinated (displayed in Fig. 2) mainly focused on how recently the vaccine had been released, the paucity of long-term data and doubt about whether the vaccine is effective. Personal views were generally similar between White and Black ethnic groups. Several respondents from White ethnic groups expressed that they felt it was too soon to have any view.

As seen in Fig. 4, across both ethnic groups, unvaccinated midwives emphasised that informed choice was needed to improve vaccine uptake. Suggestions for how to inform people included a list of ingredients, a long-term vaccination plan with regards to boosters, and more research and unbiased information. Amongst unvaccinated respondents of Black ethnicity, the theme of exploring individual concerns and not pushing people to have the vaccine was more prominent.

## Discussion

To our knowledge, this study is the first qualitative and quantitative study to explore COVID-19 vaccine uptake rates and reasons for vaccine hesitancy within midwives in the UK.

This study found that Black midwives were 4 times less likely to have had the COVID-19 vaccine than their White midwifery colleagues. The delta variant was found to have 11 times higher mortality amongst unvaccinated people, and so lower rates of vaccination amongst Black staff members risks entrenching existing inequalities in COVID-19 morbidity and mortality [18–21]. The government report on disparities in COVID-19 published in December 2021 demonstrated that during the first wave, rates of infection were higher in people from Black ethnic groups when compared with White, whereas in the third wave infection rates were higher in White ethnic groups [22]. However, hospitalisation and mortality remained disproportionately high for Black and ethnically minoritised groups which is likely attributable to lower vaccine uptake [23, 24].

A lower intention to be vaccinated in female individuals and those of Black ethnicity became evident from earlier studies investigating intention to be vaccinated prior to roll-out [10, 25–29]. A Cohort study in the United States (US) and UK demonstrated that individuals of Black ethnicity have the highest rate of vaccine hesitancy [21]. They also reported higher vaccine hesitancy and lower uptake in healthcare workers and in females compared to males [21]. It was suggested that hesitancy amongst healthcare workers may be associated with higher incidence of COVID-19 infection and assumed immunity, or greater concern about the lack of initial safety data, especially as they were amongst the first to be offered the vaccine [21]. A different study found pressure from employers had an association with vaccine hesitancy [15]. Similarly, the UK household longitudinal study demonstrated higher vaccine hesitancy in women compared to men and even higher hesitancy in those of Black ethnicity with an odds ratio for vaccine hesitancy of 13.42 [12].

In our analysis of unvaccinated midwives’ concerns by ethnicity, we found that only two concerns were more prominent amongst Black midwives: concerns that the vaccine contained porcine/meat products, and concerns about adverse effects of the vaccine on ethnic minorities.

Our results also demonstrate that Black ethnicity midwives were more likely to be opposed to the influenza vaccine compared to White midwives, a vaccine with more evidence on safety that has been available for a longer time. This suggests that further factors drive hesitancy amongst some Black participants towards all types of vaccines, beyond any concerns that relate to the novelty of the COVID-19 vaccine.

One such factor may be the deep-rooted mistrust of medicine and science caused by countless examples of abuse and non-consensual treatment of Black people during medical history. Black midwives were more aware of one example of such mistreatment, The Tuskegee Syphilis Study which led to the preventable death of 128 of its participants [30]. It is well evidenced that mistrust in public health officials and generational trauma caused by such historical events translates into vaccination hesitancy [31–33].

Distrust in medical institutions is also because of lived experience of institutionalised racism in healthcare which is becoming increasingly supported with evidence. A US study found that infant mortality was halved when Black neonates were cared for by Black doctors as opposed to White doctors [34]. In the UK, racial and ethnic disparities in maternal mortality are widening [35]. A recent independent enquiry into racial and ethnic disparities in maternal mortality identifies systemic bias in cases where Black women died [36]. These included the lack of nuanced care and microaggression, defined as ‘brief and commonplace daily verbal, behavioural, or environmental indignities, whether intentional or unintentional, that communicate hostile, derogatory, or negative racial slights and insults’ [37].

The increased level of concern amongst unvaccinated Black participants about adverse effects of the vaccine on ethnic minorities is another example of systemic racism. A 2016 review exploring ethnicity-specific factors related to childhood immunisation found concerns amongst parents that immunisation research was not ethnically heterogeneous [38]. Under representation of ethnically minoritised people in research has been reported in UK trials due to factors such as biased recruitment, language barriers and general inaccessibility to research in deprived areas [39–41].

Addressing distrust in healthcare institutions and public health programmes is a gradual and nuanced process, involving rebuilding trust and good relationships with Black and ethnically minoritised people. There must be sustained scrutiny of systemic racism and diversified research which provides more transparency through trusted sources [41, 42]. Recommendations from the UK’s National Institute for Health Research’s INCLUDE (Innovations in Clinical Trial Design and Delivery for underserved groups) Project include involving communities in trial design and holding required activities such as follow up in easily accessible buildings within minoritised communities [42].

With regards to protecting pregnant women and people, it has been demonstrated that a significant proportion of COVID-19 inpatients acquire infection in hospital, through healthcare worker to patient transmission [43]. Improving vaccine uptake amongst midwives is expected to have a protective effect in preventing nosocomial infection of COVID-19 in pregnant people and their partners.

Midwives can be particularly influential when counselling pregnant women and people [44]. The Royal College of Obstetrician and Gynaecologists (RCOG) states that people are expected to discuss the benefits and risks of having the vaccine with their healthcare professional, which is usually their midwife, and reach a joint decision based on individual circumstances.

We therefore believed it to be important to establish the specific type and degree of concerns that all midwives had regarding the COVID-19 vaccine, regardless of vaccination status. This would enable tailored education to improve understanding of the COVID-19 vaccine, aiming to alleviate midwives’ concerns and so improve vaccine advocacy in their patient care. The need for more information was recurrent theme amongst all participants. Although resources are being published by the NHS, qualitative responses from the survey highlighted that many midwives desired more transparency in information breakdowns with clear statistics as opposed to general information. Recent reports have highlighted concerns regarding unequal access to resources that aid decision making in individuals of Black ethnicity [45, 46]. Our study established that information resources were similar between the two ethnic groups, with the most frequently occurring information resources originating from the government, the television and work newsletters. Therefore, these mediums constitute the most effective means in which we can maximise the delivery of information.

The most prominent concerns which need to be addressed were regarding the speed at which the vaccine was developed and worries about long-term effects. We must reassure individuals that although the long-term effects of the vaccine are unknown, it is unlikely that long-term side effects will arise as mRNA vaccines have been studied since the 1960s. It is also important to promote, particularly considering midwives influence on women of a reproductive age, that there is complete lack of evidence for any effect on fertility, or any feasible mechanism via which this could occur. Since the start of this study there is now much more safety data available for the safety of vaccination in pregnancy which will aid this.

Pregnant women are worse affected by COVID-19, with those of Black and minoritised ethnicities being disproportionately affected [7]. Unless we urgently tackle concerns and improve vaccine uptake amongst midwives, it is likely that the vaccine hesitancy highlighted in our study population may have an indirect effect on the pregnant women and people’s views. This could potentially exacerbate inequity in vaccine uptake between ethnicities in the pregnant population, and contribute to existing inequalities in maternal morbidity and mortality.

A limitation of our study is that of selection and representation bias, as it presents the views of those that are more likely to engage with the survey taking process. It is possible that only individuals with a particular interest or strong opinion of COVID-19 responded to the survey, thus introducing reporting bias. Although a large enough number of midwives responded to our survey to allow comparison between White and Black ethnicity midwives, the number was not large enough to allow the comparison and presentation of data for other ethnic groups that were less well represented in our population sample. Our study presents a valuable snapshot of midwives’ views on COVID-19 vaccination at this given time. However, it does not allow assessment of possible changes and shifts in opinion that may arise over time, for example due to new information presented to the respondents. We plan to repeat the survey in 6 months, to observe any changes in opinion following interventions such as targeted education programs. This will also give us an opportunity to investigate views towards the implementation of mandatory vaccination if this goes ahead as currently planned.

## Conclusions

Midwives play a vital role in building and sustaining vaccine confidence in the pregnant population. Unvaccinated pregnant women– in particular Black women- are currently one of the highest risk groups for morbidity and mortality from COVID-19. Targeted education and individualised support to alleviate midwives’ concerns highlighted in this study may improve vaccine positivity amongst midwifery staff and address discrepancy in vaccine uptake between White and Black ethnicity midwives. This is likely to translate into reduced vaccine hesitancy in pregnant women and people at a population level. Any intervention must happen alongside constant national effort to build trusting relationships between healthcare providers and Black and ethnically minoritised individuals and communities.

## Data Availability

The datasets used and/or analysed during the current study are available from the corresponding author on reasonable request.

## Abbreviations

BSUH: Brighton and Sussex University Hospitals NHS Trust
COVID-19: Coronavirus disease 2019
SARS-CoV-2: Severe acute respiratory syndrome coronavirus-2
WHO: World Health Organisation
PHE: Public Health England
US: United States
UK: United Kingdom
NHS: National Health Service
JCVI: Joint Committee on vaccination and immunization
RCOG: Royal College of Obstetrician and Gynaecologists (RCOG)

## References

[1] Liu SL, Saif L (2020). Emerging viruses without Borders: the Wuhan coronavirus. Viruses..

[2] Wang L, Wang Y, Ye D, Liu Q. Since January 2020 Elsevier has created a COVID-19 resource centre with free information in English and Mandarin on the novel coronavirus COVID- 19 . The COVID-19 resource centre is hosted on Elsevier Connect , the company ’ s public news and information . 2020;(January).

[3] Public Health England. Disparities in the risk and outcomes of COVID-19. PHE Publ 2020;89. Available from: https://www.gov.uk/government/publications/covid-19-review-of-disparities-in-risks-and-outcomes

[4] Shields A, Faustini SE, Perez-Toledo M, Jossi S, Aldera E, Allen JD (2020). SARS-CoV-2 seroprevalence and asymptomatic viral carriage in healthcare workers: a cross-sectional study. Thorax..

[5] Shah ASV, Wood R, Gribben C, Caldwell D, Bishop J, Weir A, et al. Risk of hospital admission with coronavirus disease 2019 in healthcare workers and their households: Nationwide linkage cohort study. BMJ. 2020;371. 10.1136/bmj.m3582 PMC7591828 33115726

[6] British Medical Association. COVID-19 : The risk to BAME doctors. https://www.bma.org.uk/advice-and-support/covid-19/your-health/covid-19-the-risk-to-bame-doctors Accessed 1 February 2022

[7] Knight M, Bunch K, Vousden N, Morris E, Simpson N, Gale C, et al. Characteristics and outcomes of pregnant women admitted to hospital with confirmed SARS-CoV-2 infection in UK: national population based cohort study. BMJ. 2020;369. 10.1136/bmj.m2107 PMC7277610 32513659

[8] MacDonald NE, Eskola J, Liang X, Chaudhuri M, Dube E, Gellin B (2015). Vaccine hesitancy: definition, scope and determinants. Vaccine..

[9] NHS England. Special collection of data from Commissioned Adult Extra Corporeal Membrane Oxygenation (ECMO) for Severe Respiratory Failure Service Providers - supplementary information. https://www.england.nhs.uk/statistics/wp-content/uploads/sites/2/2021/10/Supplementary-Information-ECMO-14102021.xlsx Accessed 1 February 2022

[10] Fisher KA, Bloomstone SJ, Walder J, Crawford S, Fouayzi H, Mazor KM (2020). Attitudes toward a potential SARS-CoV-2 Vaccine : a survey of U.S. Adults Ann Intern Med.

[11] Kar P. Partha Kar: Covid-19 and ethnicity-why are all our angels white? BMJ 2020;369(May):m1804. Available from: 10.1136/bmj.m1804 10.1136/bmj.m1804 32371436

[12] Robertson E, Reeve KS, Niedzwiedz CL, Moore J, Blake M, Green M, et al. Predictors of COVID-19 vaccine hesitancy in the UK household longitudinal study. Brain Behav Immun. 2021. 10.1016/j.bbi.2021.03.008 PMC7946541 33713824

[13] MBRACE-UK. Saving Lives, Improving Mothes’ Care. Lessons learned to inform maternity care from the UK and Ireland Confidential Enquiries into Maternal Deaths and Morbidity 2017–19. 2021.

[14] Buchan SA, Chung H, Brown KA, Austin PC, Fell DB, et al. Effectiveness of COVID-19 vaccines against Omicron or Delta infection. medRxiv. 2022:2021–12. 10.1001/jamanetworkopen.2022.32760 PMC9500552 36136332

[15] Bell S, Clarke RM, Ismail SA, Ojo-Aromokudu et al. COVID-19 vaccination beliefs, attitudes, and behaviours among health and social care workers in the UK: a mixed-methods study. PLoS One 2022;17(1):e0260949. 10.1371/journal.pone.0260949 PMC8786153 35073312

[16] NHS England. Vaccination as a condition of deployment for healthcare workers. 2022.

[17] Royal College of Nursing. COVID-19 and mandatory vaccination. https://www.rcn.org.uk/get-help/rcn-advice/covid-19-and-mandatory-vaccination Access 1 February 2022

[18] Collie S, Champion J, Moultrie H, Bekker LG, Gray G. Effectiveness of BNT162b2 vaccine against omicron variant in South Africa. N Engl J Med. 2021. 10.1056/NEJMc2119270 PMC8757569 34965358

[19] Apea VJ, Wan YI, Dhairyawan R, Puthucheary ZA, Pearse RM, Orkin CM, et al. Ethnicity and outcomes in patients hospitalised with COVID-19 infection in East London: an observational cohort study. BMJ Open 2021;11(1):1–11. 10.1136/bmjopen-2020-042140 PMC7813387 33455936

[20] Martin CA, Patel P, Goss C, Jenkins DR, Price A, Barton L, et al. Demographic and occupational determinants of anti-SARS-CoV-2 IgG seropositivity in hospital staff. J Public Health (Bangkok). 2020:1–12. 10.1093/pubmed/fdaa199 PMC7717317 33200200

[21] Nguyen LH, Joshi AD, Ph D, Drew DA, Ph D, Ph D, et al. Racial and ethnic differences in COVID-19 vaccine hesitancy and uptake. 2021;1–49.

[22] HM Government. Final report on progress to address COVID-19 health inequalities. Race disparity unit, cabinet Office. 2021. https://assets.publishing.service.gov.uk/government/uploads/system/uploads/attachment_data/file/1038338/2021-12-03_Final_COVID-19_disparities_report___updated_3_Dec.pdf. Accessed 21 Jan 2022.

[23] Vaccine Coverage. OpenSafely, NHS England. 2022. https://reports.opensafely.org/reports/vaccine-coverage/#summarychart. Accessed 13 Jan 2022.

[24] Dyer O. Covid-19: unvaccinated face 11 times risk of death from delta variant, CDC data show. BMJ. 2021. 10.1136/bmj.n2282 34531181

[25] Williams L, Flowers P, McLeod J, Young D, Rollins L. Social patterning and stability of COVID-19 vaccination acceptance in Scotland: will those most at risk accept a vaccine? medRxiv. 2020. 10.3390/vaccines9010017 PMC7824425 33406762

[26] Latkin CA, Dayton L, Yi G, Colon B, Kong X. Mask usage, social distancing, racial, and gender correlates of COVID-19 vaccine intentions among adults in the US. PLoS One. 2021;16(2 February):1–11. Available from: 10.1371/journal.pone.0246970 10.1371/journal.pone.0246970 PMC7886161 33592035

[27] Momplaisir F, Haynes N, Nkwihoreze H, Nelson M, Werner RM, Jemmott J. Understanding Drivers of Coronavirus Disease 2019 Vaccine Hesitancy Among Blacks. Clin Infect Dis. 2021;(Xx Xxxx):1–6. 10.1093/cid/ciab102 PMC7929035 33560346

[28] Allen JD, Abuelezam NN, Rose R, Fontenot HB. Factors associated with the intention to obtain a COVID-19 vaccine among a racially/ethnically diverse sample of women in the USA. 19. 10.1093/tbm/ibab014 PMC8083705 33769536

[29] Salmon DA, Dudley MZ, Brewer J, Kan L, Gerber Je et al. COVID-19 vaccination attitudes, values and intentions among United States adults prior to emergency use authorization. Vaccine. 2021;39(19):2698–2711. 10.1016/j.vaccine.2021.03.034 PMC7988387 33781601

[30] Baker SM, Brawley OW, Marks LS (2005). Effects of untreated syphilis in the negro male, 1932 to 1972: a closure comes to the Tuskegee study, 2004. Urology..

[31] Bell S, Clarke RM, Ismail SA, Ojo-Aromokudu O, Naqvi H, et al. COVID-19 vaccination beliefs, attitudes, and behaviours among health and social care workers in the UK: a mixed-methods study. medRxiv. 2021. 10.1371/journal.pone.0260949 PMC8786153 35073312

[32] Kadambari S, Vanderslott S. Lessons about COVID-19 vaccine hesitancy among minority ethnic people in the UK. Lancet Infect Dis. 2021. 10.1016/S1473-3099(21)00404-7. 10.1016/S1473-3099(21)00404-7 PMC8352490 34384531

[33] Reverby SM. Listening to narratives from the Tuskegee syphilis study. Lancet. 2011. 10.1016/s0140-6736(11)60663-6. 10.1016/s0140-6736(11)60663-6 21591285

[34] Greenwood BN, Hardeman RR, Huang L, Sojourner A (2020). Physician–patient racial concordance and disparities in birthing mortality for newborns. Proc Natl Acad Sci.

[35] Knight M, Bunch K, Kenyon S, Tuffnell D, Kurinczuk JJ (2020). A national population-based cohort study to investigate inequalities in maternal mortality in the United Kingdom, 2009-17. Paediatr Perinat Epidemiol.

[36] Knight M, Bunch K, Vousden N, Banerjee A, Cox P, Cross-Sudworth F (2022). A national cohort study and confidential enquiry to investigate ethnic disparities in maternal mortality. EClin Med..

[37] Sue DW, Capodilupo CM, Torino GC, Bucceri JM, Holder A, Nadal KL (2007). Racial microaggressions in everyday life: implications for clinical practice. Am Psychol.

[38] Forster AS, Rockliffe L, Chorley AJ, Marlow LA, Bedford H (2017). Ethnicity-specific factors influencing childhood immunisation decisions among black and Asian minority ethnic groups in the UK: a systematic review of qualitative research. J Epidemiol Community Health.

[39] Smart A, Harrison E (2017). The under-representation of minority ethnic groups in UK medical research. Ethnicity Health.

[40] Treweek S, Forouhi NG, Narayan KV, Khunti K (2020). COVID-19 and ethnicity: who will research results apply to?. Lancet.

[41] Gill PS, Plumridge G, Khunti K, Greenfield S (2013). Under-representation of minority ethnic groups in cardiovascular research: a semi-structured interview study. Fam Pract.

[42] UK National Insitute for Health Research: Increasing participation of Black Asian and Minority Ethnic (BAME) groups in health and social care research. 2018. https://arc-em.nihr.ac.uk/clahrcs-store/increasing-participation-black-asian-and-minority-ethnic-bame-groups-health-and-social. Accessed: 31 Jan 2022.

[43] Moynan D, Cagney M, Dhuthaigh AN, Foley M, Salter A, Reidy N, et al. The role of healthcare staff COVID-19 screening in infection prevention & control. J Infect. 2020;81(3):e53–4. Available from: 10.1016/j.jinf.2020.06.057. 10.1016/j.jinf.2020.06.057 PMC7316459 32593656

[44] Ishola DA, Permalloo N, Cordery RJ, Anderson SR (2013). Midwives’ influenza vaccine uptake and their views on vaccination of pregnant women. J Public Heal (United Kingdom).

[45] Hanif W, Ali SN, Patel K, Khunti K (2020). Cultural competence in covid-19 vaccine rollout. BMJ..

[46] Corbie-Smith G. Vaccine hesitancy is a scapegoat for structural racism. JAMA Heal Forum 2021 Mar 25 [cited 2021 Apr 20];2(3):e210434. Available from: https://jamanetwork.com/10.1001/jamahealthforum.2021.0434 36218456

